# Histological remission in inflammatory bowel disease and risk of adverse pregnancy outcomes: A nationwide study

**DOI:** 10.1016/j.eclinm.2022.101722

**Published:** 2022-11-07

**Authors:** Karl Mårild, Jonas Söderling, Olof Stephansson, Jordan Axelrad, Jonas Halfvarson, SWIBREG Study Group, Gabriella Bröms, Jan Marsal, Ola Olén, Jonas F. Ludvigsson

**Affiliations:** a Department of Paediatrics, Institute of Clinical Sciences, Sahlgrenska Academy, Gothenburg, Sweden; b Department of Paediatrics, Queen Silvia Children's Hospital, Gothenburg, Sweden; c Department of Medical Epidemiology and Biostatistics, Karolinska Institutet, Solna, Sweden; d Clinical Epidemiology Division, Department of Medicine Solna, Karolinska Institutet, Stockholm, Sweden; e Inflammatory Bowel Disease Centre at NYU Langone Health, Division of Gastroenterology, Department of Medicine, NYU Grossman School of Medicine, New York, NY, USA; f Department of Gastroenterology, Faculty of Medicine and Health, Örebro University, Örebro, Sweden; g Department of Gastroenterology, Danderyd hospital, Stockholm, Sweden; h Department of Gastroenterology, Skåne University Hospital, Lund, Sweden; i Immunology Section, Lund University, Lund, Sweden; j Sachs' Children and Youth Hospital, Stockholm South General Hospital, Stockholm, Sweden; k Department of Clinical Science and Education Södersjukhuset, Karolinska Institutet, Stockholm, Sweden; l Department of Paediatrics, Örebro University Hospital, Örebro, Sweden; m Department of Medicine, Columbia University College of Physicians and Surgeons, New York, NY, USA

**Keywords:** Histology, Population-based, Pregnancy

## Abstract

**Background:**

Inflammatory bowel disease (IBD) has been linked to adverse pregnancy outcomes, but it is unclear how risks vary by histological activity.

**Methods:**

We performed a nationwide study of Swedish women diagnosed with IBD 1990–2016 and a pre-pregnancy (<12 months) colorectal biopsy with vs. without histological inflammation (1223 and 630 births, respectively). We also examined pregnancy outcomes in 2007–2016 of women with vs. without clinically active IBD (i.e., IBD-related hospitalization, surgery, or medication escalation) <12 months before pregnancy (2110 and 4993 births, respectively). Accounting for smoking, socio-demographics, and comorbidities, generalized linear models estimated adjusted risk ratios (aRRs) for preterm birth (<37 gestational weeks) and small-for-gestational age (SGA, <10th percentile weight for age).

**Findings:**

Of infants to women with vs. without histological inflammation, 9.6% (n = 117) and 6.5% (n = 41) were preterm, respectively (aRR = 1.46; 95%CI = 1.03–2.06). Histological inflammation was associated with preterm birth in ulcerative colitis (UC) (aRR = 1.64; 95%CI = 1.07–2.52), especially extensive colitis (aRR = 2.37; 95%CI = 1.12–5.02), but not in Crohn's disease (aRR = 0.99; 95%CI = 0.55–1.78). Of infants to women with vs. without histological inflammation, 116 (9.6%) and 56 (8.9%), respectively, were SGA (aRR = 1.09; 95%CI = 0.81–1.47). Clinically active disease before pregnancy was linked to preterm birth (aRR = 1.42; 95%CI = 1.20–1.69), but not to SGA birth (aRR = 1.13; 95%CI = 0.96–1.32). Finally, of infants to women without clinical activity, histological inflammation was not significantly associated with preterm birth (aRR = 1.20; 95%CI = 0.68–2.13).

**Interpretation:**

Histological and clinical activity in IBD, especially in UC, were risk factors for preterm birth. Further research is needed to determine the importance of pre-pregnancy histological activity in women without clinically-defined disease activity.

**Funding:**

The Swedish Society of Medicine.

**Research in context**

**Evidence before this study**

On June 23, 2022, we searched PubMed using the following search string: ("inflammatory bowel diseases"[All Fields] OR "crohn disease"[All Fields] OR "colitis, ulcerative"[All Fields]) AND ("histology"[All Fields] OR "histological"[All Fields] OR "microscopic"[All Fields]) AND "pregnancy"[All Fields]". The search generated 21 studies; however, none of the studies concerned histological activity of inflammatory bowel disease (IBD) to pregnancy outcomes. We then searched PubMed for articles published between Jan 1, 2000, until June 23, 2022, using the following search string: ("inflammatory bowel diseases"[All Fields] OR "crohn disease"[All Fields] OR "colitis, ulcerative"[All Fields]) AND ("activity"[All Fields] OR "flare"[All Fields] OR "relapse"[All Fields]) AND "pregnancy"[All Fields]" This second search generated 250 studies, of which a large proportion were outside the scope of this study concerning aspects of IBD management and drug safety during pregnancy.

**Added value of this study**

Based on almost 2000 births of women with IBD with a pre-pregnancy colorectal biopsy, this nationwide study found an almost 50% increased risk of preterm birth of infants to women with histological inflammation in IBD, particularly in ulcerative colitis. Histological inflammation in IBD was not linked to other adverse pregnancy outcomes.

**Implications of all available evidence**

Our data support recommendations to objectively assess IBD remission status before conception, to optimise management and prevent adverse outcomes of pregnancy and IBD.

## Introduction

Inflammatory bowel diseases (IBDs), represented mainly by Crohn's disease (CD) and ulcerative colitis (UC), are chronic inflammatory conditions, impacting patients' quality of life and imposing high societal costs. Clinical remission and mucosal healing during endoscopic evaluation are key therapeutic targets of IBD.^1^ However, microscopic disease activity can persist without clinical and endoscopic activity and has been suggested as an independent risk factor for disease flares.^2^ Histological remission (i.e., the absence of inflammation and ulceration/erosion) is therefore increasingly recognized as a potential therapeutic goal in IBD, particularly in UC.^3^

IBD diagnosis usually coincides with childbearing years.^4^ Concerns related to adverse pregnancy outcomes are therefore common, and several studies have linked IBD to adverse pregnancy outcomes.^5^, ^6^, ^7^, ^8^ In recent years, guidelines have underlined the importance of disease control to reduce pregnancy risks.^9^^,^^10^ However, these recommendations originate from data using clinical proxies for increased disease activity, such as IBD-related hospitalization, surgery, or medication, where adverse effects on pregnancy are difficult to separate from disease phenotype and patient characteristics. Despite the recent increasing appreciation of histological activity in IBD, its association with pregnancy outcomes is unknown.

Using a nationwide IBD cohort with histopathology data,^11^ we sought to investigate whether histological inflammation in IBD predicts adverse pregnancy outcomes. Our secondary aim was to determine the risk of adverse pregnancy outcomes in IBD women with and without evidence of clinical disease activity.

## Methods

### Study sample: Women with IBD and their infants

We defined IBD as having either ≥2 International Classification of Disease (ICD) codes in the National Patient Register (NPR)^12^ or ≥1 ICD code and ≥1 relevant colorectal histopathology code from the nationwide ESPRESSO cohort^11^ (see Supplementary Table S1). The Swedish NPR contains both inpatient and non-primary outpatient care.^12^ The ESPRESSO cohort consists of all computerized gastrointestinal histology reports from Sweden's 28 pathology departments. This IBD definition has a 93–95% consistency with a clinical diagnosis of IBD.^13^^,^^14^ Moreover, we specified IBD as UC, CD, or, if a distinction between subtypes could not be made, as IBD-unclassified (IBD-U). As previously described,^14^, ^15^, ^16^ patients with UC and CD were characterized according to IBD-related surgery and the extent and location of the disease at diagnosis (Supplementary Tables S2 and S3). While we lacked data on the endoscopic appearance of IBD, we describe the estimated endoscopic inflammation of IBD using a validated prediction algorithm.^17^

We identified 7374 women diagnosed with IBD in 1990–2016 who, after diagnosis, gave birth to 11,374 children in 1990–2016 (Supplementary Tables S4 and S5). 1990–2016 was chosen as a reasonable trade-off between recency (with relevance to modern IBD), and the need for a sufficient number of pregnancies to allow us to detect clinically meaningful differences.

### Exposures: Histological inflammation and clinical disease activity

#### We examined two measures of IBD activity

I. *Our primary exposure was histological inflammation* (vs. histological remission) documented 0–<12 months (≤365 days) before the start of pregnancy. In line with recent consensus reports,^3^^,^^18^ histological remission was defined through ileal-colorectal histopathology (SNOMED) codes M00100/M00110 (normal mucosa),^11^ the absence of SNOMED codes for inflammation (acute or chronic), and ulceration/erosion (Supplementary Table S6). Histological inflammation was defined as ≥1 SNOMED codes for ileal-colorectal inflammation (acute or chronic) or ulceration/erosion, meaning that the histological inflammation vs. remission was defined based on the worst histological appearance across all intestinal segments. Specific scoring systems for histopathology of IBD^3^^,^^18^ are not routinely used in Sweden.
II. *Our secondary exposure was clinically active IBD* (vs. quiescent IBD). Motivated by a previous study,^19^ we defined “clinically active IBD” as IBD-related surgery, hospitalization, or at least one budesonide or corticosteroid dispensing (either systemic or locally acting), initiation of immunomodulators (e.g., thiopurines) or biologics 0–<12 months before pregnancy (initiation of therapy was defined as dispensing/administration without record of such use in the previous 6 months). Data were retrieved from three high-quality national registers: the NPR,^12^ the Swedish Prescribed Drug Register,^20^ and the Swedish Quality Register for IBD (SWIBREG).^21^ Medications were identified using the Anatomical Therapeutic Chemical pharmaceutical classification system (Supplementary Table S7). Disease without these features was defined as “quiescent IBD.” The Prescribed Drug Register includes prospectively recorded data on all filled drug prescriptions since July 2005,^20^ and for this study, captured throughout 2016. To differentiate prevalent from incident therapy by using a minimum 6-month capture period of prescribed drugs, analyses of disease activity were restricted to births from October 1, 2007, throughout 2016 (e.g., a child born in 2010 to a woman diagnosed with IBD in 1992 is included in this analysis).

### Adverse pregnancy outcomes

High-quality data on pregnancy outcomes were retrieved from the Medical Birth Register, which contains information on all births in Sweden since 1973.^22^^,^^23^ This register also records maternal age at delivery, parity, self-reported smoking (yes/no), and body mass index (BMI) at early pregnancy. Covariates were categorized as shown in Table 1 (data on smoking was missing in 4.5% and on BMI in 8.8% of pregnancies).

**Table 1:** Characteristics of births to women diagnosed with inflammatory bowel disease (IBD) in 1990–2016 giving birth in 1990–2016 (by histological inflammation) and in October 2007–2016 (by proxies for clinical IBD activity).

Variable	Births in 1990–2016			Births in Oct 2007–2016		
	Overall	Biopsy <12 months before pregnancy^a^		Overall	Clinical IBD activity <12 months before pregnancy^b^	
		Histological inflammation	Histological remission		Clinically active	Clinically quiescent
N women with IBD	7374	1164	611	5106	1817	3849
**N deliveries**	11,374	1223	630	7103	2110	4993
Parity						
1	5073 (44.6%)	587 (48.0%)	299 (47.5%)	3127 (44.0%)	925 (43.8%)	2202 (44.1%)
2	4440 (39.0%)	456 (37.3%)	244 (38.7%)	2816 (39.6%)	871 (41.3%)	1945 (39.0%)
≥3	1861 (16.4%)	180 (14.7%)	87 (13.8%)	1160 (16.3%)	314 (14.9%)	846 (16.9%)
Smoking in early pregnancy						
Yes	725 (6.4%)	87 (7.1%)	39 (6.2%)	317 (4.5%)	116 (5.5%)	201 (4.0%)
No	10,139 (89.1%)	1085 (88.7%)	559 (88.7%)	6526 (91.9%)	1917 (90.9%)	4609 (92.3%)
Missing data	510 (4.5%)	51 (4.2%)	32 (5.1%)	260 (3.7%)	77 (3.6%)	183 (3.7%)
BMI in early pregnancy						
Median (IQR)	23.3 (21.2–26.1)	23.5 (21.2–26.2)	22.8 (21.0–25.2)	23.4 (21.3–26.2)	23.3 (21.2–26.3)	23.4 (21.3–26.2)
Living with partner						
Yes	10,349 (91.0%)	1122 (91.7%)	567 (90.0%)	6512 (91.7%)	1941 (92.0%)	4571 (91.5%)
No	146 (1.3%)	17 (1.4%)	10 (1.6%)	79 (1.1%)	31 (1.5%)	48 (1.0%)
Missing data	879 (7.7%)	84 (6.9%)	53 (8.4%)	512 (7.2%)	138 (6.5%)	374 (7.5%)
Level of education						
≤9 years	770 (6.8%)	86 (7.0%)	54 (8.6%)	419 (5.9%)	150 (7.1%)	269 (5.4%)
10–12 years	4882 (42.9%)	546 (44.6%)	248 (39.4%)	2651 (37.3%)	841 (39.9%)	1810 (36.3%)
≥13 years	5685 (50.0%)	587 (48.0%)	327 (51.9%)	4013 (56.5%)	1115 (52.8%)	2898 (58.0%)
Missing data	37 (0.3%)	4 (0.3%)	1 (0.2%)	20 (0.3%)	4 (0.2%)	16 (0.3%)
Country of birth, n (%)						
Nordic	10,534 (92.6%)	1120 (91.6%)	566 (89.8%)	6497 (91.5%)	1913 (90.7%)	4584 (91.8%)
Non-Nordic	840 (7.4%)	103 (8.4%)	64 (10.2%)	606 (8.5%)	197 (9.3%)	409 (8.2%)
Comorbidity, n (%)^c^						
Diabetes (type 1, type 2, gestational)	204 (1.8%)	21 (1.7%)	12 (1.9%)	142 (2.0%)	47 (2.2%)	95 (1.9%)
Hypertension	106 (0.9%)	11 (0.9%)	4 (0.6%)	79 (1.1%)	24 (1.1%)	55 (1.1%)
Asthma	520 (4.6%)	50 (4.1%)	28 (4.4%)	424 (6.0%)	188 (8.9%)	236 (4.7%)
Autoimmune disease	846 (7.4%)	90 (7.4%)	51 (8.1%)	690 (9.7%)	261 (12.4%)	429 (8.6%)
Any of the above	1525 (13.4%)	153 (12.5%)	87 (13.8%)	1197 (16.9%)	458 (21.7%)	739 (14.8%)
Alcohol-related disorders	99 (0.9%)	5 (0.4%)	12 (1.9%)	83 (1.2%)	32 (1.5%)	51 (1.0%)
Year of IBD diagnosis, n (%)						
1990–1999	3427 (30.1%)	373 (30.5%)	143 (22.7%)	1002 (14.1%)	266 (12.6%)	736 (14.7%)
2000–2009	6393 (56.2%)	587 (48.0%)	347 (55.1%)	4547 (64.0%)	1323 (62.7%)	3224 (64.6%)
2010–2016	1554 (13.7%)	263 (21.5%)	140 (22.2%)	1554 (21.9%)	521 (24.7%)	1033 (20.7%)
Age (years) at IBD diagnosis						
Median (IQR)	25.2 (21.3–29.2)	26.5 (22.1–30.4)	25.6 (21.7–29.6)	24.5 (20.5–28.5)	24.2 (20.4–28.2)	24.6 (20.5–28.6)
Year of delivery, n (%)						
1990–1999	981 (8.6%)	170 (13.9%)	27 (4.3%)	0	0	0
2000–2009	4746 (41.7%)	496 (40.6%)	229 (36.3%)	1456 (20.5%)	460 (21.8%)	996 (19.9%)
2010–2016	5647 (49.6%)	557 (45.5%)	374 (59.4%)	5647 (79.5%)	1650 (78.2%)	3997 (80.1%)
Age (years) at delivery						
Median (IQR)	31.2 (28.0–34.5)	30.3 (27.3–33.8)	30.9 (27.6–34.6)	31.5 (28.2–34.8)	30.9 (27.5–34.4)	31.7 (28.6–34.9)
Time (years) from IBD diagnosis to delivery						
Median (IQR)	5.1 (2.4–8.8)	2.0 (1.1–6.7)	3.9 (1.4–9.5)	6.3 (3.1–10.0)	5.7 (2.6–9.5)	6.5 (3.3–10.2)
IBD subtype						
CD	4031 (35.4%)	334 (27.3%)	214 (34.0%)	2427 (34.2%)	841 (39.9%)	1586 (31.8%)
UC	6927 (60.9%)	831 (67.9%)	393 (62.4%)	4400 (61.9%)	1171 (55.5%)	3229 (64.7%)
IBD-U	416 (3.7%)	58 (4.7%)	23 (3.7%)	276 (3.9%)	98 (4.6%)	178 (3.6%)
Montreal classification CD^d^						
L1/L3/LX	3018 (74.9%)	231 (69.2%)	164 (76.6%)	1995 (82.2%)	695 (82.6%)	1300 (82.0%)
L2	612 (15.2%)	58 (17.4%)	40 (18.7%)	387 (15.9%)	141 (16.8%)	246 (15.5%)
L missing/ICD code before 1997	401 (9.9%)	45 (13.5%)	10 (4.7%)	45 (1.9%)	5 (0.6%)	40 (2.5%)
Montreal classification UC^d^						
E1/E2	2660 (38.4%)	297 (35.7%)	152 (38.7%)	1901 (43.2%)	362 (30.9%)	1539 (47.7%)
E3	2290 (33.1%)	315 (37.9%)	147 (37.4%)	1691 (38.4%)	605 (51.7%)	1086 (33.6%)
EX	1380 (19.9%)	136 (16.4%)	78 (19.8%)	762 (17.3%)	193 (16.5%)	569 (17.6%)
E missing/ICD code before 1997	597 (8.6%)	83 (10.0%)	16 (4.1%)	46 (1.0%)	11 (0.9%)	35 (1.1%)
Endoscopy^e^						
Ever before pregnancy	10,637 (93.5%)	1223 (100%)	630 (100%)	6777 (95.4%)	2077 (98.4%)	4700 (94.1%)
0–<12 months	3330 (29.3%)	1223 (100%)	630 (100%)	2065 (29.1%)	1050 (49.8%)	1015 (20.3%)
0–<6 months	1944 (17.1%)	740 (60.5%)	378 (60.0%)	1197 (16.9%)	651 (30.9%)	546 (10.9%)
During pregnancy	1614 (14.2%)	249 (20.4%)	88 (14.0%)	1005 (14.1%)	350 (16.6%)	655 (13.1%)
Endoscopic inflammation <12m before pregnancy (Ref. 17)						
Low inflammation	493 (4.3%)	43 (3.5%)	432 (68.6%)	328 (4.6%)	129 (6.1%)	199 (4.0%)
High inflammation	1988 (17.5%)	1180 (96.5%)	198 (31.4%)	1142 (16.1%)	613 (29.1%)	529 (10.6%)
Clinically active IBD^b^						
Births from October 2007 until 2016	7103 (62.4%)	691 (56.5%)	436 (69.2%)	7103 (100%)	2110 (100%)	4993 (100%)
IBD-related hospitalization	1346 (11.8%)	337 (27.6%)	110 (17.5%)	748 (10.5%)	748 (35.5%)	0
IBD-related surgery	295 (2.6%)	90 (7.4%)	23 (3.7%)	147 (2.1%)	147 (7.0%)	0
IBD-related drugs	2018 (28.4%)	366 (53.0%)	176 (40.4%)	1808 (25.5%)	1808 (85.7%)	0
Corticosteroid	1497 (21.1%)	293 (42.4%)	134 (30.7%)	1370 (19.3%)	1370 (64.9%)	0
Budesonide	395 (5.6%)	67 (9.7%)	59 (13.5%)	351 (4.9%)	351 (16.6%)	0
Immunomodulators	556 (7.8%)	101 (14.6%)	41 (9.4%)	469 (6.6%)	469 (22.2%)	0
Biologics	162 (2.3%)	35 (5.1%)	14 (3.2%)	148 (2.1%)	148 (7.0%)	0
Biopsy (ileum-colorectum)^a^						
Ever before conception	10,512 (92.4%)	1223 (100%)	630 (100%)	6698 (94.3%)	2064 (97.8%)	4634 (92.8%)
0–<12 months	2481 (21.8%)	1223 (100%)	630 (100%)	1470 (20.7%)	742 (35.2%)	728 (14.6%)
0–<6 months	1381 (12.1%)	684 (55.9%)	357 (56.7%)	809 (11.4%)	420 (19.9%)	389 (7.8%)
During pregnancy	693 (6.1%)	85 (7.0%)	29 (4.6%)	387 (5.4%)	103 (4.9%)	284 (5.7%)
Time (months) from biopsy to start of pregnancy						
Mean (SD)		5.7 (3.4)	5.6 (3.4)			
Median (IQR)		5.7 (2.7–8.5)	5.3 (2.8–8.4)			
Biopsy 0–<12 months before pregnancy						
Births from October 2007 until 2016	1853 (16.3%)	1223 (100%)	0	1127 (15.9%)	547 (25.9%)	580 (11.3%)
Inflammation	1223 (66.0%)	1223 (100%)	0	691 (61.3%)	373 (68.2%)	318 (54.8%)
Remission	630 (34.0%)	0	630 (100%)	436 (38.7%)	174 (31.8%)	262 (45.2%)

BMI, body mass index (kg/m2); CD, Crohn's disease; IBD-U, IBD-unclassified; IQR, interquartile range; SD, standard deviation; UC, ulcerative colitis.

a Histopathology reports, as recorded in the ESPRESSO cohort,^11^ originating from the ileum or colorectum (topographic codes T65, T67, and T68) with SNOMED code for inflammation and normal histology, respectively, as detailed in Supplementary Table S6.

b Clinical IBD activity defined as IBD-related surgery, hospitalization, or IBD medication as detailed in Supplementary Table S7.

c Definitions of comorbidities are listed in Supplementary Table S8.

d Extent and location of disease at the time of diagnosis as detailed in Supplementary Table S2.

e Upper or lower endoscopy as recorded in the National Patient Register (NPR), The Swedish Quality Register for IBD or ESPRESSO histology data.

Our primary outcomes considered the risk of preterm birth (i.e., <37 gestational weeks; spontaneous, medically induced, and overall) and small for gestational age (SGA, <10th percentile of birth weight for gestational age and sex) birth. Gestational age was determined by second-trimester ultrasonography (since 1990, performed in some 95% of pregnant women in Sweden) or the last menstrual period as reported at the first maternity health care visit. Birth date and gestational age data were used to define the start of pregnancy.

Secondary pregnancy outcomes included very preterm birth (<32 gestational weeks), low birth weight (<2500 g), low 5-min Apgar score (<7), induction of labour, instrumental delivery, caesarean section (elective, emergency, and overall), stillbirth (data available since 2008 and classified as foetal deaths ≥22 gestational weeks), gestational diabetes and pre-eclampsia as defined in Supplementary Table S8.

### Other data

As detailed in Supplementary Table S8 and Table 1, we retrieved NPR data on selected medical conditions diagnosed before delivery, which may confound the relationship between IBD and pregnancy outcomes. Socioeconomic differences strongly influence pregnancy outcomes and may be linked to IBD. Consequently, we retrieved data on the highest attained education level as a proxy for socioeconomic status. Another potential confounder was country of birth. Finally, we used NPR data to describe the proportion of births where the mother had a record of alcohol-related diseases and disorders.^24^

### Patient and public involvement

Several of the researchers of this study care for patients with IBD. However, in this register-based study no patients were directly involved in the design, recruitment or conduct of the study.

### Statistical analysis

We used generalised linear models to estimate risk ratios (RRs) and their 95% confidence intervals (CIs) for adverse pregnancy outcomes of women with histological inflammation (vs. histological remission) and clinically active IBD (vs. quiescent IBD), respectively, 0–<12 months before the start of pregnancy. All analyses accounted for maternal clustering due to multiple or repeated births and were further adjusted for maternal age at delivery, parity, education level, country of birth, comorbidities, early-pregnancy smoking status, and BMI.

We present complete case analyses, which ignored observations with incomplete covariate data. However, in sensitivity analyses for the outcomes preterm birth and SGA we used multiple imputation^25^ to account for missing BMI and smoking data.

Our primary outcomes of preterm birth and SGA were examined by IBD subtype (UC, CD, IBD-U) and the extent and location of disease at diagnosis. The risks of preterm birth and SGA were also analysed stratified by year of IBD diagnosis (1990–1999, 2000–2009, 2010–2016), duration of IBD diagnosis at delivery (<2, ≥2 years), age at delivery (15–24, 25–29, 30–34, 35–39, 40–44 years), parity (nulliparous, parous), maternal education level (≤9, 10–12, ≥13 years of education), and country of birth (Nordic, Other).

#### We performed the following sensitivity analyses

**Exposure window** restricted to 0–<6 months before or during pregnancy (i.e., start of pregnancy until birth).
**Histological inflammation in women without clinically active IBD.** We determined the possible incremental effect of residual histological inflammation in women without clinical disease activity. Restricting data to births from October 1, 2007, through 2016, we estimated the risk of preterm birth and SGA in women with histological inflammation, but without clinical disease activity (i.e., “clinical activity = 0” and “inflammation = 0” [reference category] vs. “clinical activity = 0” but “inflammation = 1”).
**Outcome definition.** To test the robustness of our findings, SGA was also defined as below −2 standard deviations of weight for age and sex.

### Post-hoc analysis

*Stratified analyses of histological inflammation and preterm birth by use of corticosteroids:* Restricted to births between October 2007 and December 2016 for which Prescribed Drug Registry data were available, we stratified our analysis on histological inflammation (vs histological remission) and preterm birth by pre-pregnancy use of corticosteroids.

Data were analysed using SAS version 9.4 (SAS Institute, Inc).

The Stockholm Ethics Review Board approved the study and waived the requirement of informed consent.

### Role of the funding source

The funding sources did not influence any aspect of the study (its design, the collection, analysis, and interpretation of data), its writing or the decision to submit it for publication. JFL, JS and OO had full access to all the data in the study. All authors accepted responsibility to submit the paper for publication.

## Results

### Patient characteristics: Histological inflammation vs. histological remission

This study included 7374 women diagnosed with IBD between 1990 and 2016 who, after their diagnosis, gave birth to 11,374 children (up until 2016). Restricting our sample to women with ileal-colorectal biopsies collected 0–<12 months before pregnancy, there were 1223 births to 1164 women with histological inflammation and 630 births to 611 women with histological remission (Table 1). Median 2 (range 1–13) biopsies from colorectum and terminal ileum were obtained in each patient.

Overall, 35.4% of the children were born by women with CD, 60.9% with UC, and 3.7% with IBD-U. Women with histological inflammation more often had a high degree of endoscopic inflammation and a higher risk of IBD-related surgery or hospitalization in the year before pregnancy than those in histological remission (Table 1). Until delivery, the median disease duration was shorter in the inflammation group (2.0 years) than those without histological inflammation (3.9 years). Pre-pregnancy histological inflammation was more common in births in the 1990s than after 2000 (Table 1). However, smoking habits, BMI, parental cohabitation, and education level were similar in women with and without histological inflammation (Table 1).

### Main results: Histological inflammation of IBD and risk of preterm birth and SGA

The preterm birth rate was 7.8% (n = 887/11,322) in the overall sample of IBD women. Among those with histologic inflammation within 12 months before pregnancy, 9.6% (n = 117) of births were preterm compared to 6.5% (n = 41) in women with no inflammation corresponding to an almost 50% increased risk of preterm birth (RR 1.46; 95%CI 1.04–2.05) and represents one extra event per 32 births in women with histological inflammation. Adjustment for maternal age, smoking habits, BMI, education level, and comorbidity exerted negligible impact on results (adjusted (a)RR 1.46 (95%CI 1.03–2.06; Table 2). Risks were numerically higher for medically induced (aRR 1.88; 95%CI 0.96–3.67) than spontaneous preterm births (aRR 1.31; 95%CI 0.86–1.98). In sensitivity analyses the aRRs for preterm birth were similar for histological inflammation within 6 months before pregnancy (aRR 1.41; 95%CI 0.90–2.21) and histological inflammation during pregnancy (aRR 1.38; 95%CI 0.72–2.64).

**Table 2:** Risk of any preterm birth according to histological inflammation of inflammatory bowel disease (IBD) < 12 months before pregnancy.

Group	Live births in all women	Live births in women with		Risk ratio^c^ (95% CI)	Adjusted risk ratio^d^ (95% CI)
		Histological inflammation^a^	Histological remission^b^		
**N live births**	11,322	1218	627		
Overall, any preterm birth	887 (7.8%)	117 (9.6%)	41 (6.5%)	1.46 (1.04–2.05)	1.46 (1.03–2.06)
Medically induced^e^	328 (2.9%)	41 (3.4%)	11 (1.8%)	1.93 (0.99–3.75)	1.88 (0.96–3.67)
Spontaneous	559 (4.9%)	76 (6.2%)	30 (4.8%)	1.31 (0.86–1.97)	1.31 (0.86–1.98)
**Stratified analyses**					
IBD subtype					
CD	301 (7.5%)	23 (6.9%)	16 (7.5%)	0.92 (0.51–1.68)	0.99 (0.55–1.78)
UC	550 (8.0%)	88 (10.6%)	24 (6.1%)	1.70 (1.11–2.60)	1.64 (1.07–2.52)
IBD-U	36 (8.7%)	6 (10.3%)	1 (4.3%)	2.31 (0.31–17.49)	-
Montreal classification CD^f^					
L1/L3/LX	240 (8.0%)	18 (7.8%)	14 (8.6%)	0.92 (0.47–1.79)	1.15 (0.58–2.28)
L2	38 (6.2%)	3 (5.2%)	1 (2.5%)	–	–
Montreal classification UC^f^					
E1/E2	160 (6.0%)	17 (5.7%)	9 (5.9%)	0.97 (0.44–2.12)	–
E3	221 (9.7%)	40 (12.8%)	8 (5.5%)	2.28 (1.12–4.66)	2.37 (1.12–5.02)
EX	114 (8.3%)	16 (11.8%)	5 (6.4%)	1.83 (0.70–4.80)	1.66 (0.63–4.37)
Year of IBD diagnosis, n (%)					
1990–1999	286 (8.4%)	40 (10.8%)	13 (9.1%)	1.19 (0.65–2.17)	1.13 (0.62–2.04)
2000–2009	476 (7.5%)	55 (9.4%)	20 (5.8%)	1.61 (0.99–2.62)	1.57 (0.96–2.57)
2010–2016	125 (8.1%)	22 (8.4%)	8 (5.8%)	1.48 (0.67–3.24)	-
Time (years) from IBD diagnosis to delivery					
<2 years	225 (9.3%)	58 (9.5%)	13 (5.8%)	1.68 (0.93–3.01)	1.63 (0.92–2.88)
≥2 years	662 (7.4%)	59 (9.7%)	28 (7.0%)	1.38 (0.90–2.13)	1.35 (0.87–2.11)
Age at delivery					
15–24 years	105 (9.8%)	12 (8.1%)	4 (7.3%)	1.38 (0.41–4.71)	-
25–29 years	286 (8.1%)	41 (9.5%)	14 (6.6%)	1.44 (0.80–2.58)	1.47 (0.83–2.62)
30–34 years	301 (7.1%)	35 (8.8%)	16 (7.3%)	1.21 (0.69–2.13)	1.80 (0.83–3.91)
35–39 years	161 (7.8%)	24 (11.7%)	7 (6.0%)	1.93 (0.86–4.35)	1.91 (0.84–4.33)
40–44 years	34 (8.5%)	5 (14.3%)	0	-	-
Parity					
Nulliparous	488 (9.7%)	58 (9.9%)	22 (7.4%)	1.35 (0.84–2.16)	1.41 (0.88–2.26)
Parous	399 (6.4%)	59 (9.3%)	19 (5.8%)	1.61 (0.98–2.66)	1.63 (0.99–2.69)
Level of education					
≤9 years	67 (8.8%)	10 (11.8%)	4 (7.4%)	1.58 (0.52–4.78)	1.70 (0.45–6.45)
10–12 years	402 (8.3%)	52 (9.6%)	14 (5.7%)	1.67 (0.95–2.95)	1.64 (0.93–2.90)
≥13 years	415 (7.3%)	55 (9.4%)	23 (7.0%)	1.31 (0.83–2.07)	1.35 (0.85–2.13)
Country of birth, n (%)					
Nordic	816 (7.8%)	104 (9.3%)	35 (6.2%)	1.49 (1.03–2.16)	1.50 (1.03–2.16)
Non-Nordic	71 (8.5%)	13 (12.7%)	6 (9.4%)	1.36 (0.54–3.40)	–
**Sensitivity analyses**					
Modified exposure window					
0–<6 months before pregnancy		63 (9.7%)	24 (7.0%)	1.38 (0.88–2.16)	1.41 (0.90–2.21)
During pregnancy		49 (13.0%)	9 (9.3%)	1.34 (0.72–2.48)	1.38 (0.72–2.64)

CI, confidence interval; CD, Crohn's disease; IBD-U, IBD-unclassified; UC, ulcerative colitis.

a Histological inflammation was defined as having an ileal-colorectal histology report with ≥1 histopathology (SNOMED) codes for inflammation (Supplementary Table S6).

b Histological remission was defined through SNOMED codes M00100/M00110 (normal mucosa) and no presence of SNOMED codes for inflammation.

c Clustered on the identity of the woman.

d Clustered on the identity of the woman and adjusted for maternal age at delivery, parity, smoking in early pregnancy, body mass index (BMI) in early pregnancy, education, and comorbidity (any diabetes, hypertension, chronic autoimmune disease, and asthma; Supplementary Table S8). The model failed to converge in specific analyses (denoted by “–”).

e Causes for medically induced preterm birth include, among others, antepartum haemorrhage and pre-eclampsia.

f Extent and location of disease at diagnosis as detailed in Supplementary Table S2.

Excess risks of preterm birth were confined to women with histological inflammation in UC (aRR 1.64; 95%CI 1.07–2.52), and especially elevated in those with extensive colitis (Montreal classification E3 of UC, aRR 2.37; 95%CI 1.12–5.02). In contrast, histological inflammation in CD was not associated with preterm birth (aRR 0.99; 95%CI 0.55–1.78). While the absolute risks of preterm birth were highest in nulliparous women, young women (15–24 years), and those with low education level (≤9 years) (Table 2), the RRs by histological activity were otherwise relatively consistent across subgroups of patient characteristics (Table 2).

Women with pre-pregnancy histological inflammation gave birth to 116 (9.6%) SGA infants and those without to 56 (8.9%) (aRR 1.09; 95%CI 0.81–1.47) (overall, SGA was observed in 9.6% [n = 1083/11,322] of infants to women with IBD). Neither inflammation 0–6 months before pregnancy (aRR 0.83; 95%CI 0.54–1.25) nor inflammation during pregnancy (aRR 1.21; 95%CI 0.58–2.52) was significantly associated with SGA (Table 3). Histological inflammation was also not significantly associated with SGA within patient subgroups (Table 3).

**Table 3:** Risk of small for gestational age (SGA, <10th percentile of birth weight by age) according to histological inflammation of inflammatory bowel disease (IBD) < 12 months before pregnancy.

Group	Live births in all women	Live births in women with		Risk ratio^c^ (95% CI)	Adjusted risk ratio^d^ (95% CI)
		Histological inflammation^a^	Histological remission^b^		
**N live births**^e^	11,322	1218	627		
Overall, SGA (<10 percentile)	1083 (9.6%)	116 (9.6%)	56 (8.9%)	1.06 (0.78–1.43)	1.09 (0.81–1.47)
SGA < −2SD	323 (2.9%)	36 (3.0%)	16 (2.6%)	1.16 (0.65–2.07)	–
**Stratified analyses**					
IBD subtype					
CD	404 (10.1%)	35 (10.5%)	22 (10.4%)	1.01 (0.64–1.60)	0.96 (0.60–1.54)
UC	639 (9.3%)	77 (9.3%)	31 (7.9%)	1.17 (0.79–1.74)	1.18 (0.80–1.74)
IBD-U	40 (9.7%)	4 (6.9%)	3 (13.0%)	0.57 (0.14–2.42)	–
Montreal classification CD^f^					
L1/L3/LX	293 (9.8%)	22 (9.6%)	16 (9.9%)	0.97 (0.53–1.79)	1.06 (0.57–1.95)
L2	65 (10.6%)	6 (10.3%)	5 (12.5%)	1.00 (0.99–1.00)	–
Montreal classification UC^f^					
E1/E2	228 (8.6%)	21 (7.1%)	15 (9.9%)	0.72 (0.38–1.36)	0.67 (0.35–1.27)
E3	201 (8.9%)	29 (9.3%)	13 (9.0%)	1.04 (0.56–1.93)	1.39 (0.76–2.56)
EX	134 (9.8%)	13 (9.6%)	2 (2.6%)	3.69 (0.87–15.68)	–
Year of IBD diagnosis, n (%)					
1990–1999	386 (11.4%)	46 (12.5%)	14 (9.8%)	1.20 (0.72–1.99)	1.20 (0.71–2.04)
2000–2009	562 (8.8%)	52 (8.9%)	34 (9.9%)	0.90 (0.60–1.36)	1.02 (0.69–1.53)
2010–2016	135 (8.7%)	18 (6.9%)	8 (5.8%)	1.19 (0.53–2.68)	1.10 (0.47–2.58)
Time (years) from IBD diagnosis to delivery					
<2 years	238 (9.9%)	57 (9.4%)	12 (5.3%)	1.77 (0.97–3.23)	1.70 (0.91–3.18)
≥2 years	845 (9.5%)	59 (9.7%)	44 (11.0%)	0.89 (0.62–1.27)	0.94 (0.66–1.34)
Age at delivery					
15–24 years	138 (13.0%)	17 (11.5%)	5 (9.1%)	3.66 (1.15–11.64)	1.33 (0.50–3.57)
25–29 years	348 (9.9%)	46 (10.7%)	19 (9.0%)	1.19 (0.72–1.97)	1.14 (0.70–1.87)
30–34 years	370 (8.7%)	32 (8.1%)	19 (8.7%)	0.93 (0.54–1.60)	0.99 (0.58–1.69)
35–39 years	185 (9.0%)	20 (9.8%)	9 (7.8%)	1.00 (1.00–1.00)	–
40–44 years	42 (10.5%)	1 (2.9%)	4 (16.0%)	0.18 (0.02–1.50)	–
Parity					
Nulliparous	692 (13.7%)	72 (12.4%)	34 (11.4%)	1.08 (0.74–1.59)	1.17 (0.80–1.71)
Parous	391 (6.2%)	44 (7.0%)	22 (6.7%)	1.01 (0.63–1.64)	0.87 (0.53–1.44)
Level of education					
≤9 years	118 (15.5%)	10 (11.8%)	10 (18.5%)	0.63 (0.34–1.18)	–
10–12 years	494 (10.2%)	56 (10.4%)	25 (10.2%)	1.01 (0.65–1.56)	1.01 (0.65–1.56)
≥13 years	465 (8.2%)	50 (8.5%)	21 (6.4%)	1.32 (0.81–2.15)	1.41 (0.86–2.30)
Country of birth, n (%)					
Nordic	971 (9.3%)	107 (9.6%)	47 (8.4%)	1.14 (0.83–1.57)	1.17 (0.86–1.61)
Non-Nordic	112 (13.4%)	9 (8.8%)	9 (14.1%)	0.63 (0.26–1.49)	0.61 (0.22–1.67)
**Sensitivity analyses**					
Modified exposure window					
0–<6 months before pregnancy		52 (8.0%)	33 (9.6%)	0.83 (0.55–1.26)	0.83 (0.54–1.25)
During pregnancy		41 (10.9%)	8 (8.2%)	1.32 (0.64–2.73)	1.21 (0.58–2.52)

CI, confidence interval; CD, Crohn's disease; IBD-U, IBD-unclassified; SD, standard deviation; UC, ulcerative colitis.

SGA defined as birthweights <10th percentile (or < -2SD) for gestational age and sex of all singleton births in 1983–2010 in Sweden.

a Histological inflammation was defined as having an ileal-colorectal histology report with ≥1 histopathology (SNOMED) codes for inflammation (Supplementary Table S6).

b Histological remission was defined through SNOMED codes M00100/M00110 (normal mucosa) and no presence of SNOMED codes for inflammation.

c Clustered on the identity of the woman.

d Clustered on the identity of the woman and adjusted for maternal age at delivery, parity, smoking in early pregnancy, body mass index (BMI) in early pregnancy, education, and comorbidity (any diabetes, hypertension, chronic autoimmune disease, and asthma; Supplementary Table S8). The model failed to converge in specific analyses (denoted by “–”).

e Missing data for birth weight, all women with IBD n = 24 (0.2%), histological inflammation n = 4 (0.3%) and histological remission n = 1 (0.2%).

f Extent and location of disease at diagnosis as detailed in Supplementary Table S2.

### Patient characteristics: Clinically active IBD vs. quiescent IBD

From October 1, 2007, and until 2016, approximately one third (n = 2110) of the study infants were born to women with clinically active IBD and two thirds (n = 4993) were born to women with clinically quiescent IBD. Almost 90% of those with clinically active IBD were identified through their use of IBD-related drugs (Table 1). Characteristics of women with active IBD were similar to women with histological inflammation (Table 1). Two thirds (68.2%; n = 373/547) and 54.8% (n = 318/580) of women with and without clinically active IBD, respectively, had histological inflammation within 12 months before pregnancy (Table 1).

### Clinically active IBD and risk of preterm birth and SGA

In women with clinically active IBD 0–<12 months before pregnancy, 9.8% (n = 206) of births were preterm compared to 6.5% (n = 325) of births to women with clinically quiescent IBD (aRR 1.42; 95%CI 1.20–1.69). There was a higher risk of preterm birth in women with clinical IBD activity *during pregnancy* (aRR 1.71; 95%CI 1.45–2.02; Table 4). In line with the results for histological inflammation, the highest aRRs for preterm birth were observed in women with active extensive UC (Montreal classification E3 [pancolitis], aRR 1.84 [95%CI 1.37–2.48]; Montreal E1/E2 [proctitis/left-sided UC], aRR 1.09 [95%CI 0.69–1.74]). Clinically active IBD was associated with both medically induced preterm birth (aRR 1.49; 95%CI 1.12–1.99) and spontaneous preterm birth (aRR 1.41; 95%CI 1.13–1.76) (Table 4).

**Table 4:** Risk of any preterm birth according to clinically active inflammatory bowel disease (IBD) < 12 months before pregnancy.

Group	Live births in all women	Live births in women with		Risk ratio^b^ (95% CI)	Adjusted risk ratio^c^ (95% CI)
		Clinically active IBD^a^	Clinically quiescent IBD^a^		
**N live births**	7064	2094	4970		
Overall, any preterm birth	531 (7.5%)	206 (9.8%)	325 (6.5%)	1.45 (1.23–1.72)	1.42 (1.20–1.69)
Medically induced^d^	203 (2.9%)	81 (3.9%)	122 (2.5%)	1.53 (1.15–2.03)	1.49 (1.12–1.99)
Spontaneous	328 (4.6%)	125 (6.0%)	203 (4.1%)	1.44 (1.15–1.79)	1.41 (1.13–1.76)
**Stratified analyses**					
IBD subtype					
CD	173 (7.2%)	70 (8.4%)	103 (6.5%)	1.28 (0.95–1.73)	1.22 (0.90–1.66)
UC	335 (7.7%)	128 (11.0%)	207 (6.4%)	1.64 (1.33–2.04)	1.62 (1.31–2.01)
IBD-U	23 (8.4%)	8 (8.2%)	15 (8.5%)	1.00 (0.44–2.24)	0.98 (0.43–2.22)
Montreal classification CD^e^					
L1/L3/LX	148 (7.5%)	61 (8.8%)	87 (6.7%)	1.29 (0.93–1.80)	1.22 (0.87–1.72)
L2	22 (5.7%)	9 (6.4%)	13 (5.3%)	1.21 (0.53–2.76)	1.33 (0.66–2.71)
Montreal classification UC^e^					
E1/E2	108 (5.7%)	23 (6.4%)	85 (5.5%)	1.12 (0.71–1.77)	1.09 (0.69–1.74)
E3	159 (9.5%)	82 (13.7%)	77 (7.2%)	1.84 (1.37–2.48)	1.84 (1.37–2.48)
EX	65 (8.6%)	22 (11.5%)	43 (7.6%)	1.52 (0.93–2.48)	1.44 (0.87–2.39)
Year of IBD diagnosis, n (%)					
1990–1999	73 (7.3%)	25 (9.5%)	48 (6.6%)	1.42 (0.88–2.28)	1.41 (0.89–2.23)
2000–2009	333 (7.4%)	130 (9.9%)	203 (6.3%)	1.51 (1.22–1.88)	1.47 (1.18–1.82)
2010–2016	125 (8.1%)	51 (9.8%)	74 (7.2%)	1.32 (0.94–1.86)	1.35 (0.96–1.90)
Time (years) from IBD diagnosis to delivery					
<2 years	102 (9.0%)	40 (9.8%)	62 (8.6%)	1.13 (0.77–1.66)	1.14 (0.77–1.69)
≥2 years	429 (7.2%)	166 (9.8%)	263 (6.2%)	1.53 (1.27–1.86)	1.49 (1.23–1.81)
Age at delivery					
15–24 years	61 (9.9%)	28 (11.8%)	33 (8.8%)	1.23 (0.75–2.00)	1.17 (0.71–1.92)
25–29 years	155 (7.4%)	58 (8.6%)	97 (6.9%)	1.23 (0.91–1.67)	1.18 (0.87–1.60)
30–34 years	179 (6.6%)	71 (9.7%)	108 (5.5%)	1.76 (1.31–2.36)	1.75 (1.30–2.36)
35–39 years	112 (8.1%)	40 (11.0%)	72 (7.0%)	1.53 (1.05–2.23)	1.46 (1.00–2.14)
40–44 years	24 (8.6%)	9 (10.8%)	15 (7.7%)	1.30 (0.60–2.80)	-
Parity					
Nulliparous	284 (9.1%)	104 (11.3%)	180 (8.2%)	1.38 (1.10–1.73)	1.36 (1.08–1.72)
Parous	247 (6.2%)	102 (8.7%)	145 (5.2%)	1.65 (1.29–2.11)	1.56 (1.21–2.00)
Level of education					
≤9 years	32 (7.8%)	13 (8.8%)	19 (7.2%)	1.24 (0.63–2.44)	1.16 (0.59–2.31)
10–12 years	217 (8.2%)	89 (10.6%)	128 (7.1%)	1.44 (1.10–1.87)	1.44 (1.11–1.87)
≥13 years	279 (7.0%)	104 (9.4%)	175 (6.1%)	1.51 (1.20–1.91)	1.48 (1.17–1.88)
Country of birth, n (%)					
Nordic	483 (7.5%)	184 (9.7%)	299 (6.5%)	1.43 (1.20–1.71)	1.40 (1.17–1.68)
Non-Nordic	48 (8.0%)	22 (11.3%)	26 (6.4%)	1.68 (0.97–2.91)	1.69 (0.97–2.92)
**Sensitivity analyses**					
Modified exposure window					
0–<6 months before pregnancy		136 (10.5%)	395 (6.8%)	1.49 (1.24–1.79)	1.44 (1.20–1.74)
During pregnancy		268 (10.8%)	263 (5.8%)	1.79 (1.51–2.11)	1.71 (1.45–2.02)

CI, confidence interval; CD, Crohn's disease; IBD-U, IBD-unclassified; UC, ulcerative colitis.

a Clinical IBD activity defined as IBD-related surgery, hospitalization, or IBD medication as detailed in Supplementary Table S7, or without these features regarded as clinically quiescent IBD.

b Clustered on the identity of the woman.

c Clustered on the identity of the woman and adjusted for maternal age at delivery, parity, smoking in early pregnancy, body mass index (BMI) in early pregnancy, education, and comorbidity (any diabetes, hypertension, chronic autoimmune disease, and asthma; Supplementary Table S8). The adjusted model failed to converge in specific analyses (denoted by “–”).

d Causes for induced preterm delivery include, among others, antepartum haemorrhage and pre-eclampsia.

e Extent and location of disease at diagnosis as detailed in Supplementary Table S2.

Some 203 (9.7%) and 416 (8.4%) infants born to women with and without clinically active IBD <12 months before pregnancy, respectively, were SGA (aRR = 1.13; 95%CI 0.96–1.32). Active IBD before pregnancy was also not significantly associated with SGA within patient subgroups of varying education levels, ages, etc (Supplementary Table S9). However, there was a modestly increased SGA risk in children to women with active IBD *during pregnancy* (aRR 1.17 [95%CI 1.01–1.37]; Supplementary Table S9).

#### Preterm birth and SGA related to histological inflammation in clinically quiescent IBD

In a sensitivity analysis of 726 children to women with clinically quiescent IBD, the presence of histological inflammation (vs. no inflammation) was not significantly associated with preterm birth (aRR 1.20; 95%CI 0.68–2.13; Table 5) (for reference, the main analysis for inflammation yielded an aRR of 1.46 [95%CI 1.03–2.06]). Histological inflammation in clinically quiescent IBD was not associated with SGA (aRR 0.98; 95%CI 0.60–1.60).

**Table 5:** Risk of any preterm birth and small for gestational age (SGA, <10th percentile of birth weight by age) in inflammatory bowel disease (IBD) patients with *clinically quiescent disease* <12 months before pregnancy.

Group	Live births in all women	Live births in women with clinically quiescent disease a and		Risk ratio^d^ (95% CI)	Adjusted risk ratio^e^ (95% CI)
		Histological inflammation^b^	Histological remission^c^		
N	7064	317	409		
Preterm birth	531 (7.5%)	23 (7.3%)	23 (5.6%)	1.20 (0.68–2.12)	1.20 (0.68–2.13)
N with birth weight	7057	316	408		
SGA <10th percentile	181 (2.6%)	24 (7.6%)	35 (8.6%)	0.86 (0.53–1.42)	0.98 (0.60–1.60)

CI, confidence interval.

SGA defined as birthweights <10th percentile (or < -2SD) for gestational age and sex of all singleton births in 1983–2010 in Sweden.

a No record of IBD-related hospitalization, surgery, or medical escalation as detailed in Supplementary Table S7.

b Histological inflammation was defined as having an ileal-colorectal histology report with ≥1 histopathology (SNOMED) codes for inflammation (Supplementary Table S6).

c Histological remission was defined through SNOMED codes M00100/M00110 (normal mucosa) and no presence of SNOMED codes for inflammation.

d Clustered on the identity of the woman.

e Clustered on the identity of the woman and adjusted for maternal age at delivery, parity, smoking in early pregnancy, body mass index (BMI) in early pregnancy, education, and comorbidity (any diabetes, hypertension, chronic autoimmune disease, and asthma; Supplementary Table S8).

#### Secondary pregnancy outcomes

For our secondary outcomes, caesarean section was more prevalent in women with (31.8%) vs. without (24.3%) clinically active IBD (aRR 1.25; 95%CI 1.17–1.34); Supplementary Table S10). However, caesarean section was not significantly linked to histologic inflammation (25.8% [inflammation] vs. 23.0% [remission]; aRR 1.10; 95%CI 0.93–1.30; Supplementary Table S11). Pre-eclampsia was not linked to histologic inflammation or clinical IBD activity (Supplementary Tables S10 and S11).

### Post-hoc analysis

Restricting our cohort to children born between October 2007 and December 2016 (period for which maternal data on drugs were available) we reran our analysis on histological inflammation and preterm birth while stratifying for corticosteroid use. However, these analyses yielded largely similar estimates across strata (in women with corticosteroid use: aHR = 1.30; 95% CI 0.66–2.55; in women without corticosteroid use: aHR = 1.35; 95% 0.77–2.36).

The association of histological inflammation vs histological remission and clinically active vs quiescent IBD with SGA and preterm birth were largely unchanged when adjusting for alcohol-related diseases and disorders (Supplementary Table S12) as well as when using multiple imputation to account for missing BMI and smoking covariate data (Supplementary Tables S13 and S14).

## Discussion

In this nationwide study of maternal IBD, histological inflammation was associated with preterm birth, but not other adverse pregnancy outcomes. The increased risk for preterm birth was confined to women with UC and histological inflammation, particularly extensive colitis. However, histological inflammation was not significantly linked to preterm birth in women with clinically quiescent disease. Our data support recommendations to objectively assess endoscopic and histologic disease remission before conception,^9^ to optimize management and prevent adverse outcomes of pregnancy and IBD. This is particularly important against the background that in our IBD cohort (1990–2016), two thirds of biopsied women had histological inflammation and one third had clinical disease activity within 12 months before pregnancy.

Clinical and endoscopic remission are the main therapeutic targets of IBD. However, histological remission may further improve clinical outcomes beyond these therapeutic targets, particularly in UC, where it has been proposed as a potential adjunct treatment goal.^1^^,^^3^ Less is known regarding the potential additional prognostic benefits of histological remission in CD,^18^ especially outside randomized controlled trials. Overall, there is little data on the long-term prognostic outcomes of histological remission in IBD.

This study links histological inflammation with preterm birth. Excess risks were seen in UC, but not in CD, with the highest risk increase in women with extensive colitis (aRR 2.37; 95%CI 1.12–5.02). Our contrasting UC and CD findings are consistent with previous research showing that a high degree of endoscopic inflammation was linked to hospitalization in UC patients.^17^ This association was less evident in CD.^17^ Even if histological inflammation was associated with an overall modest risk increase for preterm birth (aRR in our primary analysis was 1.46 [95%CI 1.03–2.06]), its impact on a child's health can be substantial as preterm birth is a major cause of death in children younger than 5 years.^26^^,^^27^ Preterm birth also increases the risk of health sequelae in adulthood.^28^ Recent data from the Pregnancy in Inflammatory Bowel Disease and Neonatal Outcomes study found infants to women exposed to corticosteroids during pregnancy (suggesting more active disease) to be at greater risk of serious infections at 9 and 12 months of age,^29^ potentially mediated through preterm birth.^30^

In our study, aRRs for spontaneous preterm birth were only slightly lower than preterm birth overall, indicating that this association is not merely explained by preterm labour induction. Indeed, considerable data implicate inflammation in the pathogenesis of preterm birth,^31^ further underlining the need to control histological and clinical disease activity in IBD to minimize the impact on maternal and foetal health. To what extent the association between histological inflammation and preterm birth might have been mediated through anti-inflammatory medication was beyond the scope of this study. A multi-centre study recently observed a link between corticosteroid use and preterm birth in women with IBD.^29^ However, we found the association between histological inflammation and preterm birth to be largely similar among women with or without use of corticosteroids. On the other hand, histological inflammation was not significantly linked to preterm birth in women with clinically quiescent disease suggesting that part of the association of histological appearance with preterm birth was related to disease activity.

We and others,^32^ have reported that approximately one in three IBD women have clinical disease activity soon before pregnancy and demonstrate, consistent with previous studies, that those women have an increased risk of caesarean section, especially elective caesarean section.^33^^,^^34^ The current study adds to existing literature, however, by showing that the association with caesarean section is not restricted to disease activity defined by self-reports or inpatient care^29^^,^^34^; nor is our study likely to suffer from confounding from smoking and BMI that may have hampered some studies. Guidelines recommend caesarean delivery to be primarily dictated by obstetric necessity and to limit elective caesarean section to specific IBD phenotypes (e.g., active rectal-perianal involvement or rectovaginal fistula).^9^^,^^10^ Still, women with active IBD may have been recommended caesarean delivery more often than women with quiescent disease. We could not confirm or refute a small excess risk for emergency caesarean section in women with histological inflammation (aRR 1.22 [95%CI 0.95–1.56]). This is noteworthy as an emergency, but not elective caesarean section, is usually performed for foetal rather than maternal reasons.

We exploited population-based histopathology data as an objective measure of disease activity in IBD. More than 1800 births to women with IBD biopsied <12 months before pregnancy were identified. Earlier validations have shown a positive predictive value of 93%–95% for our IBD definition,^13^^,^^14^ and the validity for the covariates used in our statistical model retrieved from the NPR is high.^12^ The prospective data collection eliminates recall bias. Through linkage to data on hospital admission, validated IBD surgery data,^16^ and medication use to estimate clinical disease activity, we could examine the association between histological inflammation and adverse pregnancy outcomes in the absence of clinical IBD activity. Other strengths include using the well-characterized data from the Medical Birth Register, and linkage to other national registers virtually guarantee a complete follow-up of individuals. Finally, we were able to calculate absolute risks (i.e., proportion of events), which are essential in planning health care and communicating risks.

This study has several limitations. As in any observational study, causality cannot be inferred. However, a study randomizing pregnant women (or women desiring pregnancy) to histological or clinical disease activity would be unethical and unlikely to be performed. Thus, while we adjusted for the potential confounding of maternal age, education level, comorbidities, smoking, BMI, and birth country, we recognize that residual confounding cannot be ruled out. Furthermore, the consistency in our findings across patient subgroups, defined by age and calendar year of diagnosis, does not support confounding by indication, i.e., an association related to the indication for biopsy (e.g., cancer surveillance, change in therapeutic management) as a sole cause of our findings.

Another limitation is the lack of data on the clinical indication, symptoms and biomarkers, that could have prompted the histological assessment of IBD. However, women with histological inflammation vs histological remission were more likely to have features of clinically defined disease activity, such as prior IBD-related surgery or hospitalization (Table 1), which suggest that the histological assessment was at least partly related to evaluation and management of disease activity. Another shortcoming is that we did not report whether pregnancy outcomes vary by endoscopic or biochemical remission data. Hence, it is unknown to what extent the association between histological inflammation and preterm birth is explained by the endoscopic appearance of IBD, although we believe that a vast majority of individuals with histological inflammation also have endoscopic inflammation. Furthermore, because histological assessment was not routinely performed before pregnancy, such data were unavailable for all pregnant women. Still, our nationwide approach is likely to have identified all eligible IBD women with a pre-pregnancy histological assessment and reduces the risk of selection bias. Also, comparisons of patient characteristics and pregnancy outcomes of all IBD women under study with those undergoing histological assessment suggest representativeness.

We acknowledge that clinical IBD activity defined by IBD-related hospitalization, surgery, or medication escalation may identify patients with moderate-to-severe disease activity. Patients without these features may not have reached complete clinical or endoscopic remission. Similarly, we recognize that routine histological assessments may not capture the full burden of IBD activity, above all in CD patients, where intramural or segmentally distributed intestinal inflammation may be misclassified as histological remission. In addition, UC patients may be histologically misclassified given that microscopic activity can vary within and across colonic segments, especially after therapy. Finally, in this nationwide study, misclassification may also be related to interobserver variability between clinical pathologists. Importantly, however, the prospective nature of this study ensures that any misclassification of histopathological data should be unrelated to pregnancy outcome. While such non-differential misclassification may attenuate risk estimates, it is unlikely to cause spurious associations. Our data originate from Sweden, a high-income country of mainly Caucasian people with a high incidence of IBD. The generalizability of our findings to other countries is unknown.

Using nationwide prospectively collected data, we showed that histological inflammation in IBD, particularly UC, was associated with an increased risk of preterm birth. While our results suggest that targeting histological remission of women with UC may reduce the risk of preterm birth, further research is needed to determine the importance of pre-pregnancy histological appearance in women without clinically defined disease activity.

## Contributors

JFL and OO designed the study, collected study data and are responsible for data integrity. JS analysed the data. JFL, JS, and OO had full access to all the data in the study and verified the underlying data for our results. KM and JFL wrote the first draft of the paper. JS, OS, JA, JH, GB, JM, and OO contributed to the writing of the paper. KM, JFL, and OO obtained funding. JFL supervised the project and is the guarantor of the article. KM, JS, OS, JA, JH, GB, JM, OO, and JFL interpreted the data, agreed with the manuscript's results and conclusions, approved the final version of the paper and accepted responsibility to submit it for publication.

## Data sharing statement

No additional data are available due to Swedish regulations.

## Declaration of interests

Dr Ludvigsson has coordinated an unrelated study on behalf of the Swedish Quality Registry for IBD (SWIBREG). That study received funding from the Janssen corporation.

Dr Olén has been PI for projects (unrelated to the current paper) at Karolinska Institutet financed by Janssen, Takeda, AbbVie, Ferring, and Pfizer grants. Karolinska Institutet has received fees for lectures (OO) and participation on advisory boards (OO) from Janssen, Ferring, Bristol Myers Squibb, Galapagos, and Takeda.

Dr Halfvarson has served as a speaker or advisory board member for AbbVie, Celgene, Celltrion, Dr. Falk Pharma and the Falk Foundation, Ferring, Galapagos, Gilead, Hospira, Janssen, MEDA, Medivir, MSD, Novartis, Olink Proteomics, Pfizer, Prometheus Laboratories Inc., Sandoz, Shire, Takeda, ThermoFisher Scientific, Tillotts Pharma, Vifor Pharma, and UCB. He has also received grant support from Janssen, MSD, and Takeda.

Dr Axelrad has received research grants from BioFire Diagnostics; consultancy fees or honorarium from BioFire Diagnostics, Abbvie, Pfizer, and Janssen; and holds U.S. patent 2012/0052124A1.

Dr. Bröms has participated in projects at Karolinska Institutet financed by grants from Janssen, Pfizer, and UCB. Lecturer for Takeda.

Dr Marsal has served as a speaker, consultant, or advisory board member for AbbVie, Bayer, Bristol-Myers Squibb, Hospira, Janssen-Cilag, Merck Sharp & Dohme, Pfizer, Sandoz, Takeda, and Union Chimique Belge and has received grant support from AbbVie, Calpro AS, Fresenius Kabi, Pfizer, SVAR Life Science, and Takeda.

The other authors report no conflicts of interest.
