# Supplementary material for: Histological remission in inflammatory bowel disease and risk of adverse pregnancy outcomes: A nationwide study
Source: eClinicalMedicine. 2022 Nov 7;53:101722. doi: 10.1016/j.eclinm.2022.101722 (PMC9716329; doi:10.1016/j.eclinm.2022.101722)
Supplement: Supplementary Material [file mmc1.pdf]

SUPPLEMENT

Histological remission in inflammatory bowel disease and risk of adverse pregnancy outcomes: A nationwide study

Karl Mårild, MD PhD, Jonas Söderling, PhD, Olof Stephansson, MD PhD, Jordan Axelrad, MD, Jonas Halfvarson, MD PhD, SWIBREG Study Group, Gabriella Bröms, MD PhD, Jan Marsal, MD PhD, Ola Olén, MD PhD, Jonas F Ludvigsson, MD PhD

Table S1. International Classification of Disease (ICD) codes and Systematized Nomenclature of Medicine (SNOMED) histology used in the definition of inflammatory bowel diseases (IBD)

	**ICD-9**	**ICD-10**	**SNOMED codes [2]**
**Swedish National Patient Register**	**1987-1996**	**1997-**	**1965-**
Ulcerative colitis (UC)	556	K51	D6255<br>*or*<br>M41, M42, M43, M44, M463, or M47
Crohn's disease (CD)	555	K50	D6216<br>*or*<br>M41, M42, M43, M44, M463, or M47
IBD unclassified (IBD-U)	UC+CD	UC + CD,<br>or K52·3	D6214<br>*or*<br>M41, M42, M43, M44, M463, or M47

[1]Topographic code T67-T68

[2]For details, please see:

Olen O, Erichsen R, Sachs MC, Pedersen L, Halfvarson J, Askling J, et al. Colorectal cancer in Crohn's disease: a Scandinavian population-based cohort study. Lancet Gastroenterol Hepatol. 2020. doi:10.1016/S2468-1253(20)30005-4

Olen O, Erichsen R, Sachs MC, Pedersen L, Halfvarson J, Askling J, et al. Colorectal cancer in ulcerative colitis: a Scandinavian population-based cohort study. Lancet. 2020;395:123-31. doi:10.1016/S0140-6736(19)32545-0

Table S2. Definitions and diagnostic codes used to define ulcerative colitis and Crohn's disease according to the Montreal classification since the start of the tenth revision of the International Classification of Diseases (ICD-10) (1997).

**Ulcerative colitis**	**Extent (E)**	**Diagnostic codes**
E1	Ulcerative proctitis	K51·2
E2	Left-sided	K51·3; K51·5
E3	Extensive (pancolitis)	K51·0
EX	Extent not defined	K51·4; K51·8; K51·9
**Crohn's disease**	**Location (L)**	**Diagnostic codes**
L1	Ileitis	K50·0
L2	Colonic	K50·1
L3/LX	Ileocolonic/location not defined	K50·8, K50·9

Table S3. Surgery codes included in the definitions of inflammatory bowel disease (IBD)-related bowel surgery and perianal surgery (since 1964).

Classification of surgical procedures	6th revision	7th revision
**Colectomy**		
**1) Subtotal colectomy with end ileostomy**		
Colectomy and ileostomy with closure of the rectum	4651	JFH10
Laparoscopic colectomy and ileostomy		JFH11
Other colectomy		JFH96
**2) Colectomy with IRA (ileorectal anastomosis)**		
Colectomy with ileorectal anastomosis	4650	JFH00
Laparoscopic colectomy with ileorectal anastomosis		JFH01
Ileorectal anastomosis		JFC40
Laparoscopic ileorectal anastomosis		JFC41
Closure of enterostomy with anastomosis to the rectum		JFG29
Closure of enterostomy with anastomosis to the colon		JFG26
**3) Partial colectomies**		
Right-sided colectomy	4641	JFB30, JFB31
Resection of the colon transversum	4643	JGB40, JFB41
Left-sided colectomy	4640	JFB43, JFB44
Resection of the sigmoid colon	4644	JFB46, JFB47
Other colon resection	4649	JFB50, JFB51
**4) Proctocolectomy with IPAA (ileal pouch-anal anastomosis)**		
Colectomy, rectal mucosectomy and ileoanal anastomosis *without* ileostomy.		JFH30
Colectomy, rectal mucosectomy and ileoanal anastomosis *and* ileostomy.		JFH33
Mucosectomy and ileoanal anastomosis after previous colectomy.	4654	JGB50
Extirpation of rectum or making of an ileoanal anastomosis after previous colectomy.		JGB60
**5) Continent ileostomy at time of colectomy**		
Proctocolectomy with continent ileostomy, "Kock"	4653	JFH40
Converting a conventional ileostomy to a continent ileostomy		JFG60
**6) Proctocolectomy**		
Proctocolectomy with ileostomy	4652	JFH20
**Other bowel surgery**	**1964-96**	**1997-**
Strictureplasty to the small bowel		JFA60
Strictureplasty to the colon		JFA63
Closure of small intestinal fistula		JFA76
Closure of colonic fistula		JFA86
Colonic and/or small bowel resection	4630, 4631, 4640-4649	JFB
Formation of stoma		JFF

Operations on intestinal stoma or reservoir		JFG
Other operation of the small bowel and/or colon	4660-4668, 4700-4739, 4790-4798	JFW96
Other laparoscopic operation of the small bowel and/or colon		JFW97
Rectal resection	4820-4828	JGB
**Perianal surgery**		
Perianal incision and drainage	4900	JHA00
Dilatation of the anal sphincter	4960	JHD00
Lay open or excision of perianal fistula	4920, 4922-4924	JHD20
Partial lay open or excision of perianal fistula (including seton placement)	4970-4971	JHD30
Completion lay open or excision of perianal fistula		JHD33
Excision of perianal fistula with advancement flap		JHD50
Occlusion of perianal fistula with collagen plug		JHD60
Occlusion of perianal fistula with fibrin glue		JHD63
Other anal or perianal surgery (e.g., examination under anaesthesia)	4999	JHW96

Table S4. Formation of study sample. Women diagnosed with inflammatory bowel disease (IBD) in 1990-2016 and who after their diagnosis gave birth in 1990-2016[1]

Inclusion/exclusion	N births	N births excluded	% births excluded	N women with IBD
Women with IBD diagnosis in 1990-2016	43 624			
Reused personal identity number	43 597	27	0%	
Death before index date	43 571	26	0%	
Giving birth after IBD diagnosis	11 904	31 667	73%	7 511
Age <15y at delivery	11 904	0	0%	
Age >44y at delivery	11 864	40	0%	
Multiple births	11 474	390	3%	
Missing gestational age	11 470	4	0%	
≥4 births for the same woman[2]	11 374	96	1%	7 374
**Total**	**11 374**	**32 250**	**74%**	**7 374**

[1] IBD diagnosed in 1990-2016 any time before birth, i.e., either before or during pregnancy; we required no time-lag from first diagnosis until delivery. [2] Excluded to improve the convergence of the models that accounted for maternal clustering due to multiple or repeated births.

Table S5. Number of births per included woman diagnosed with inflammatory bowel disease (IBD) in 1990-2016 and giving birth in 1990-2016

Birth order in the study	N births	%
1	7 374	64·8%
2	3 394	29·8%
3	606	5·3%
**Total births**	**11 374**	**100%**

Table S6. Systematized Nomenclature of Medicine (SNOMED) codes for inflammation and normal mucosa in inflammatory bowel disease

SNOMED code	Morphology
M00100	Normal
M00110	Normal
M40000	Unspecified inflammation
M40400	Pustulous inflammation, unspecified
M41000	Acute inflammation
M42100	Acute and chronic inflammation
M43000	Chronic inflammation
M43030	Ulcer, chronic inflammation
M42000	Subacute inflammation
M40460	Inflammation with pus
M40700	Inflammation with necrosis
M41700	Inflammation with necrosis, abscess
M41740	Abscess
M44000	Granulomatous inflammation
M44700	Necrotizing granulomatous inflammation
M44900	Necrotizing fibrinous inflammation
M45020	Inflammation with granulation

Table S7. Anatomical Therapeutic Chemical (ATC) codes of inflammatory bowel disease (IBD) treatment.

Drug group	Substance	ATC-code
Immune modulators	Azathioprine	L04AX0 1
	Mercaptopurine	L01BB02
	Methotrexate	L04AX03/L01BA01
Anti-tumour necrosis factor-a (TNFa) treatment	Infliximab	L04AB02 (L04AA12 before 2008)
	Adalimumab	L04AB04 (L04AA17 before 2008)
	Golimumab	L04AB06
Other biologics	Vedolizumab	L04AA33
	Ustekinumab	L04AC05
Corticosteroids, systemic	Betamethasone	H02AB01
	Dexamethasone	H02AB02
	Methylprednisolone	H02AB04
	Prednisolone	H02AB06
	Prednisone	H02AB07
	Hydrocortisone	H02AB09
	Cortisone	H02AB10
Corticosteroids, locally acting	Budesonide	A07EA06

Table S8. International Classification of Diseases (ICD) codes for the definitions of gestational diabetes, pre-eclampsia, and comorbidities.

**Diagnosis**	**ICD-9**<br>1987-1996	**ICD-10**<br>≥1997
**Maternal outcomes of pregnancy**		
Gestational diabetes	648A	O244
Pre-eclampsia	642E, 642F, 642G, 642H	O14-O15
**Comorbidities[1] for adjustment**		
Diabetes (type 1, type 2, and gestational)	250	E10-E14, O24
Hypertension	401-405	I10-I15
Asthma	493	J45
Chronic autoimmune diseases[2]	242A, 242X, 244X, 245C, 245W, 579A, 696, 709A, 714, 710A	E035, E039, E050, E055, E059, E063, E065, K900, L40, L80, M05, M06, M123, M32

[1]Comorbidities as recorded until the time of delivery (i.e., before or during pregnancy). [2] Autoimmune thyroid diseases, celiac disease, psoriasis, vitiligo, rheumatoid arthritis, and systemic lupus erythematosus.

Table S9. Risk of small for gestational age (SGA, <10th percentile of birth weight by age) according to clinically active inflammatory bowel disease (IBD) <12 months before pregnancy

Group	Live births in all included women	Live births in women with		Risk ratio[2] (95% CI)	Adjusted risk ratio[3] (95% CI)
		Clinically active IBD[1]	Clinically quiescent IBD[1]		
**N live births[4]**	7 064	2 094	4 970		
**Overall, SGA (<10 percentile)**	619 (8·8%)	203 (9·7%)	416 (8·4%)	1·16 (0·99-1·36)	1·13 (0·96-1·32)
SGA <-2SD	181 (2·6%)	57 (2·7%)	124 (2·5%)	1·09 (0·81-1·47)	1·05 (0·77-1·43)
**STRATIFIED ANALYSES**					
IBD subtype					
CD	227 (9·4%)	87 (10·4%)	140 (8·9%)	1·18 (0·92-1·52)	1·11 (0·86-1·43)
UC	369 (8·4%)	107 (9·2%)	262 (8·2%)	1·14 (0·92-1·41)	1·14 (0·92-1·41)
IBD-U	23 (8·4%)	9 (9·3%)	14 (8·0%)	1·03 (0·47-2·24)	-
Montreal classification CD[5]					
L1/L3/LX	184 (9·3%)	75 (10·9%)	109 (8·4%)	1·30 (0·99-1·71)	1·22 (0·92-1·63)
L2	39 (10·1%)	12 (8·5%)	27 (11·0%)	0·78 (0·41-1·48)	0·74 (0·40-1·38)
Montreal classification UC[5]					
E1/E2	158 (8·4%)	37 (10·3%)	121 (7·9%)	1·27 (0·89-1·81)	1·26 (0·89-1·78)
E3	134 (8·0%)	49 (8·2%)	85 (7·9%)	1·05 (0·75-1·47)	1·04 (0·74-1·47)
EX	71 (9·3%)	18 (9·4%)	53 (9·3%)	1·02 (0·62-1·66)	1·03 (0·63-1·68)
Year of IBD diagnosis, n (%)					
1990-1999	92 (9·3%)	31 (11·9%)	61 (8·3%)	1·41 (0·96-2·08)	1·39 (0·95-2·04)
2000-2009	392 (8·7%)	121 (9·2%)	271 (8·4%)	1·09 (0·89-1·34)	1·04 (0·84-1·27)
2010-2016	135 (8·7%)	51 (9·8%)	84 (8·2%)	1·21 (0·87-1·67)	1·22 (0·88-1·69)
Time from IBD diagnosis to delivery					
<2 years	97 (8·6%)	38 (9·3%)	59 (8·2%)	1·14 (0·78-1·69)	1·17 (0·79-1·73)
≥2 years	522 (8·8%)	165 (9·8%)	357 (8·4%)	1·16 (0·98-1·39)	1·12 (0·94-1·34)
Age at delivery					
15 – 24 years	71 (11·6%)	29 (12·2%)	42 (11·1%)	1·08 (0·69-1·68)	1·05 (0·68-1·63)
25 – 29 years	176 (8·4%)	68 (10·0%)	108 (7·7%)	1·30 (0·98-1·74)	1·30 (0·98-1·74)
30 – 34 years	225 (8·4%)	60 (8·2%)	165 (8·4%)	0·97 (0·73-1·30)	0·96 (0·72-1·28)
35 – 39 years	120 (8·7%)	40 (11·0%)	80 (7·8%)	1·41 (0·99-2·01)	1·29 (0·90-1·84)
40 – 44 years	27 (9·7%)	6 (7·2%)	21 (10·8%)	0·67 (0·28-1·60)	0·68 (0·29-1·58)
Parity					
Nulliparous	406 (13·1%)	127 (13·9%)	279 (12·8%)	1·09 (0·89-1·32)	1·06 (0·87-1·29)
Parous	213 (5·4%)	76 (6·5%)	137 (4·9%)	1·32 (1·01-1·73)	1·27 (0·97-1·66)
Level of education					
≤9 years	60 (14·6%)	22 (15·0%)	38 (14·3%)	1·00 (0·61-1·63)	1·04 (0·64-1·70)
10 – 12 years	242 (9·2%)	85 (10·2%)	157 (8·7%)	1·14 (0·89-1·46)	1·10 (0·86-1·42)
≥13 years	315 (7·9%)	95 (8·6%)	220 (7·6%)	1·16 (0·92-1·45)	1·15 (0·91-1·44)
Country of birth, n (%)					
Nordic	545 (8·4%)	178 (9·4%)	367 (8·0%)	1·17 (0·99-1·38)	1·13 (0·95-1·34)
Non-Nordic	74 (12·4%)	25 (12·8%)	49 (12·1%)	1·06 (0·67-1·68)	0·94 (0·61-1·46)
**SENSITIVITY ANALYSES**					
Modified exposure window					
0-<6 months before pregnancy		134 (10·4%)	485 (8·4%)	1·22 (1·02-1·47)	1·17 (0·97-1·41)
During pregnancy		254 (10·2%)	365 (8·0%)	1·27 (1·09-1·47)	1·17 (1·01-1·37)

SGA defined as birthweights <10th percentile (or <-2SD) for gestational age and sex of all singleton births in 1983-2010 in Sweden [1]Defined as IBD-related surgery, hospitalization, or IBD medication as detailed in Supplementary Table S7, or without these features regarded as clinically quiescent IBD.[2] Clustered on the identity of the woman; [3] Clustered on the identity of the woman and adjusted for age at delivery, parity, smoking, body mass index (BMI), education, and comorbidity (any diabetes, hypertension, autoimmune disease and asthma; Supplementary Table S8); [4] Missing data for birth weight, all women with IBD n=7 (0·1%), histological inflammation n=4 (0·3%) and histological remission n=1 (0·2%). [5] Extent and location of disease at diagnosis as detailed in Supplementary Table S2· CI, confidence interval; CD, Crohn's disease; IBD-U, IBD-unclassified; UC, ulcerative colitis.

Table S10. Risk of adverse pregnancy outcomes in women with inflammatory bowel disease (IBD), overall and according to clinical disease activity <12 months before pregnancy

Group	Live births in all included women	Live births in women with		Risk ratio[2] (95% CI)	Adjusted risk ratio[3] (95% CI)
		Clinically active IBD[1]	Clinically quiescent IBD[1]		
**N live births**	7 064	2 094	4 970		
Foetal growth					
Low birth weight (<2500g)					
All live births	323 (4·6%)	123 (5·9%)	200 (4·0%)	1·43 (1·15-1·78)	1·38 (1·11-1·73)
Term births (≥37 gestational weeks, GW)	84 (1·3%)	33 (1·8%)	51 (1·1%)	1·58 (1·02-2·43)	1·56 (1·00-2·44)
Other neonatal outcomes					
Apgar <7 at 5 minutes	95 (1·3%)	34 (1·6%)	61 (1·2%)	1·32 (0·87-2·01)	1·33 (0·87-2·05)
Very preterm birth (<32 GW)	61 (0·9%)	18 (0·9%)	43 (0·9%)	0·99 (0·57-1·72)	0·96 (0·56-1·64)
**N all births**	7 103	2 110	4 993		
Intrauterine foetal death					
Stillbirth	39 (0·5%)	16 (0·8%)	23 (0·5%)	1·65 (0·88-3·11)	1·49 (0·78-2·82)
Pregnancy and maternal outcomes					
Induction of labour	1 162 (16·4%)	388 (18·4%)	774 (15·5%)	1·16 (1·04-1·30)	1·14 (1·02-1·27)
Caesarean section	1 883 (26·5%)	670 (31·8%)	1 213 (24·3%)	1·22 (1·14-1·31)	1·25 (1·17-1·34)
Elective	711 (10·0%)	251 (11·9%)	460 (9·2%)	1·28 (1·11-1·48)	1·32 (1·14-1·52)
Emergency	1 172 (16·5%)	419 (19·9%)	753 (15·1%)	1·23 (1·11-1·37)	1·25 (1·13-1·38)
Instrumental delivery	519 (7·3%)	152 (7·2%)	367 (7·4%)	0·98 (0·82-1·18)	1·01 (0·85-1·22)
Gestational diabetes	77 (1·1%)	21 (1·0%)	56 (1·1%)	0·90 (0·55-1·47)	0·88 (0·54-1·44)
Pre-eclampsia	244 (3·4%)	80 (3·8%)	164 (3·3%)	1·17 (0·90-1·51)	1·18 (0·91-1·53)
Missing data					
Birth weight	7 (0·1%)	4 (0·2%)	3 (0·1%)		
Birth weight term births	5 (0·1%)	4 (0·2%)	1 (0·0%)		
Apgar score at 5 minutes	20 (0·3%)	9 (0·4%)	11 (0·2%)		

[1]Clinical IBD activity defined as IBD-related surgery, hospitalization, or IBD medication as detailed in Supplementary Table S7, or without these features regarded as clinically quiescent IBD.[2] Clustered on the identity of the woman; [3] Clustered on the identity of the woman and adjusted for maternal age at delivery, parity, smoking in early pregnancy, body mass index (BMI) in early pregnancy, education, and comorbidity (any diabetes, hypertension, chronic autoimmune disease and asthma; Supplementary Table S8). CI, confidence interval.

Table S11. Risk of adverse pregnancy outcomes in women with inflammatory bowel disease (IBD), overall and according to histological inflammation <12 months before pregnancy

Group	Live births in all included women	Live births in women with		Risk ratio[3] (95% CI)	Adjusted risk ratio[4] (95% CI)
		Histological inflammation[1]	Histological remission[2]		
**N live births**	11 322	1 218	627		
Foetal growth					
Low birth weight (<2500g)					
All live births	545 (4·8%)	75 (6·2%)	23 (3·7%)	1·67 (1·06-2·63)	1·70 (1·08-2·68)
Term births (≥37 gestational weeks, GW)	147 (1·4%)	21 (1·9%)	9 (1·5%)	1·24 (0·57-2·70)	1·42 (0·67-3·00)
Other neonatal outcomes					
Apgar <7 at 5 minutes	153 (1·4%)	11 (0·9%)	11 (1·8%)	0·52 (0·23-1·19)	-
Very preterm birth (<32 GW)	102 (0·9%)	13 (1·1%)	3 (0·5%)	2·23 (0·64-7·80)	-
**N all births**	11 374	1 223	630		
Intrauterine foetal death					
Stillbirth	52 (0·5%)	5 (0·4%)	3 (0·5%)	0·86 (0·21-3·60)	0·60 (0·15-2·34)
Pregnancy and maternal outcomes					
Induction of labour	1 638 (14·4%)	162 (13·2%)	100 (15·9%)	0·84 (0·66-1·05)	0·82 (0·65-1·03)
Caesarean section	2 944 (25·9%)	316 (25·8%)	145 (23·0%)	1·10 (0·94-1·30)	1·10 (0·93-1·30)
Elective	1 153 (10·1%)	126 (10·3%)	64 (10·2%)	1·02 (0·77-1·36)	0·98 (0·74-1·30)
Emergency	1 791 (15·7%)	190 (15·5%)	81 (12·9%)	1·19 (0·93-1·53)	1·22 (0·95-1·56)
Instrumental delivery	895 (7·9%)	91 (7·4%)	58 (9·2%)	0·82 (0·60-1·12)	0·83 (0·60-1·14)
Gestational diabetes	119 (1·0%)	15 (1·2%)	9 (1·4%)	0·86 (0·38-1·95)	-
Pre-eclampsia	397 (3·5%)	52 (4·3%)	20 (3·2%)	1·31 (0·79-2·17)	1·20 (0·72-2·00)
Missing data					
Birth weight	24 (0·2%)	4 (0·3%)	1 (0·2%)		
Birth weight term births	17 (0·2%)	1 (0·1%)	0		
Apgar score at 5 minutes	51 (0·5%)	4 (0·3%)	2 (0·3%)		

[1]Histological inflammation was defined as having an ileal-colorectal histology report with ≥1 histopathology (SNOMED) codes for inflammation (Supplementary Table S6). [2]Histological remission was defined through SNOMED codes M00100/M00110 (normal mucosa) and no presence of SNOMED codes for inflammation. [3]Clustered on the identity of the woman; [4]Clustered on the identity of the woman and adjusted for maternal age at delivery, parity, smoking in early pregnancy, body mass index (BMI) in early pregnancy, education, and comorbidity (any diabetes, hypertension, chronic autoimmune disease, and asthma; Supplementary Table S8). The model failed to converge in specific analyses (denoted by "–"); CI, confidence interval.

Table S12. Results adjusted for alcohol-related diseases and disorders (ARDD). Risk of preterm birth and small-for-gestational age (SGA, <10th percentile of birth weight by age)

Group	Live-births in all included women	Live-births in women with inflammation /active disease	Live-births in women with no inflammation /no active disease	Unadjusted risk ratio* (95% CI)	Primary adjusted risk ratio** (95% CI)	Primary + ARDD-adjusted risk ratio*** (95% CI)
Preterm birth	887 (7·8%)	117 (9·6%)	41 (6·5%)	1·46 (1·04-2·05)	1·46 (1·03-2·06)	1·47 (1·05-2·07)
SGA <10th percentile	1 083 (9·6%)	116 (9·6%)	56 (8·9%)	1·06 (0·78-1·43)	1·09 (0·81-1·47)	1·10 (0·82-1·49)
**Active disease**						
Preterm birth	531 (7·5%)	206 (9·8%)	325 (6·5%)	1·45 (1·23-1·72)	1·42 (1·20-1·69)	1·42 (1·20-1·69)
SGA <10th percentile	619 (8·8%)	203 (9·7%)	416 (8·4%)	1·16 (0·99-1·36)	1·13 (0·96-1·32)	1·13 (0·96-1·32)

*Clustered on the ID of the woman;

**Clustered on the ID of the woman and adjusted for maternal age at delivery, parity, smoking in early pregnancy, BMI in early pregnancy, education, and comorbidity (diabetes, hypertension, chronic autoimmune disease, asthma).

**Clustered on the ID of the woman and adjusted for maternal age at delivery, parity, smoking in early pregnancy, BMI in early pregnancy, education, and comorbidity (diabetes, hypertension, chronic autoimmune disease, asthma, *and ARDD*).

Table S13. **Analysis with multiple imputation for missing covariate data for body mass index (BMI) and maternal smoking. Risk of preterm birth and small-for-gestational age (SGA) of infants born in 1990-2016 to women with inflammatory bowel disease (IBD) diagnosed in 1990-2016, overall and according to pre-pregnancy histological inflammation.**

Group	Live births in all included women	Live births in women with		Risk ratio[3] (95% CI)	Adjusted risk ratio[4] (95% CI)
		Histological inflammation[1]	Histological remission[2]		
**N live births**	11 322	1 218	627		
**Any preterm birth**	887 (7·8%)	117 (9·6%)	41 (6·5%)	**1·46 (1·04-2·05)**	**1·47 (1·04-2·07)**
**SGA <10th percentile**	1 083 (9·6%)	116 (9·6%)	56 (8·9%)	**1·06 (0·78-1·43)**	**1·12 (0·83-1·51)**

[1]Histological inflammation was defined as having an ileal-colorectal histology report with ≥1 histopathology (SNOMED) codes for inflammation (Supplementary Table S6). [2]Histological remission was defined through SNOMED codes M00100/M00110 (normal mucosa) and no presence of SNOMED codes for inflammation. [3]Clustered on the identity of the woman; [4]Clustered on the identity of the woman and adjusted for maternal age at delivery, parity, smoking in early pregnancy, body mass index (BMI) in early pregnancy, education, and comorbidity (any diabetes, hypertension, chronic autoimmune disease, and asthma; Supplementary Table S8).

Table S14. Analysis with multiple imputation for missing covariate data for body mass index and smoking. Risk of preterm birth and small-for-gestational age (SGA) of infants born in October 2007 to December 2016 to women with inflammatory bowel disease (IBD) diagnosed in 1990-2016, overall and according to clinical disease activity

Group	Live births in all included women	Live births in women with		Risk ratio[2] (95% CI)	Adjusted risk ratio[3] (95% CI)
		Clinically active IBD[1]	Clinically quiescent IBD[1]		
**N live births**	7 064	2 094	4 970		
**Any preterm birth**	531 (7·5%)	206 (9·8%)	325 (6·5%)	**1·45 (1·23-1·72)**	**1·42 (1·19-1·68)**
**SGA <10th percentile**	619 (8·8%)	203 (9·7%)	416 (8·4%)	**1·16 (0·99-1·36)**	**1·13 (0·96-1·32)**

[1]Clinical IBD activity defined as IBD-related surgery, hospitalization, or IBD medication as detailed in Supplementary Table S7, or without these features regarded as clinically quiescent IBD.[2] Clustered on the identity of the woman; [3] Clustered on the identity of the woman and adjusted for maternal age at delivery, parity, smoking in early pregnancy, body mass index (BMI) in early pregnancy, education, and comorbidity (any diabetes, hypertension, chronic autoimmune disease and asthma; Supplementary Table S8). CI, confidence interval.

.
